# Prevention of migraine by supraorbital transcutaneous neurostimulation using the Cefaly® device (PREMICE): a multi-centre, randomized, sham-controlled trial

**DOI:** 10.1186/1129-2377-14-S1-P184

**Published:** 2013-02-21

**Authors:** J Schoenen, B Vandersmissen, S Jeangette, L Herroelen, M Vandenheede, P Gerard, D Magis

**Affiliations:** 1 Headache Research Unit, Belgium; 2 Department of Neurology, Belgium

## Introduction

Subjects who have frequent migraine attacks (≥ 2 / month) are in need of a preventive anti-migraine treatment. Available preventive drugs have incomplete efficacy and/or unpleasant side effects.

## Purpose

Supraorbital transcutaneous neurostimulation (STNS) has shown encouraging results for migraine prevention in pilot studies and has no side effects [1-3]. We assessed efficacy and safety of STNS in migraine prophylaxis with the Cefaly® device in a multicentre, double-blind, randomized, sham-controlled trial.

## Methods

Five Belgian tertiary headache clinics participated to the study. After a 1-month run-in, patients with ≥ 2 migraine attacks/month were randomized to verum or sham stimulation, and applied the Cefaly® device daily for 20 minutes during 3 months. Primary outcome measures were change in monthly migraine days and 50% responder rate, i.e. the percentage of subjects having a ≥ 50% reduction of monthly migraine days. Patients and enrolling neurologists were blinded from the randomization.

## Results

Sixty-seven patients were randomized and included in the intention-to-treat analysis. Between run-in and 3rd month of treatment the mean number of migraine days decreased significantly in the verum (4.88 vs 6.94; p=0.023), but not in the sham group (6.22 vs 6.54; p=0.608). The 50% responder rate was significantly greater (p=0.023) in the verum (38.1%) than in the sham group (12.1 %). Monthly migraine attacks (p=0.044), monthly headache days (p=0.041) and monthly acute anti-migraine drug intake (p=0.007) were also significantly reduced in the verum but not in the sham group. There were no adverse events in either group.

## Conclusions

STNS with the Cefaly® device is effective as a preventive therapy for migraine. The therapeutic gain (26%) is within the range of those reported for other preventive drug and non-drug anti-migraine treatments [4,5], and the safety profile is excellent.

## Conflicts of interest

LH: Allergan. JS: ATI Redwood California, St Jude Medical USA, Allergan USA, ATI USA, Medtronic USA and Cyberonics USA.
